# Challenges and Recommendations for Electronic Health Records Data Extraction and Preparation for Dynamic Prediction Modeling in Hospitalized Patients: Practical Guide and Tutorial

**DOI:** 10.2196/73987

**Published:** 2025-10-17

**Authors:** Elena Albu, Shan Gao, Pieter Stijnen, Frank E Rademakers, Bas CT van Bussel, Taya Collyer, Tina Hernandez-Boussard, Laure Wynants, Ben Van Calster

**Affiliations:** 1 Department of Development & Regeneration KU Leuven Leuven Belgium; 2 Management Information Reporting Department University Hospitals Leuven Leuven Belgium; 3 Faculty of Medicine KU Leuven Leuven Belgium; 4 Care and Public Health Research Institute Maastricht University Maastricht The Netherlands; 5 Department of Intensive Care Medicine Maastricht University Medical Centre Maastricht The Netherlands; 6 Cardiovascular Research Institute Maastricht University Maastricht The Netherlands; 7 Peninsula Clinical School, Central Clinical School Monash University Melbourne Australia; 8 National Centre for Healthy Ageing Monash University Melbourne Australia; 9 Department of Medicine Stanford University Stanford, CA United States; 10 Leuven Unit for Health Technology Assessment Research KU Leuven Leuven Belgium

**Keywords:** EHR data, ETL, data extraction, data preparation, dynamic prediction models, electronic health records, extract, transform, load

## Abstract

Dynamic predictive modeling using electronic health record data has gained significant attention in recent years. The reliability and trustworthiness of such models depend heavily on the quality of the underlying data, which is, in part, determined by the stages preceding the model development: data extraction from electronic health record systems and data preparation. In this paper, we identified over 40 challenges encountered during these stages and provided actionable recommendations for addressing them. These challenges are organized into 4 categories: cohort definition, outcome definition, feature engineering, and data cleaning. This comprehensive list serves as a practical guide for data extraction engineers and researchers, promoting best practices and improving the quality and real-world applicability of dynamic prediction models in clinical settings.

## Background

Predictive modeling using electronic health record (EHR) data has become increasingly important in enhancing patient outcomes through real-time risk detection and timely interventions. However, the effectiveness of these models is heavily reliant on the quality and structure of the underlying data, which are influenced by the processes of data extraction and preparation. While recent advancements in artificial intelligence and machine learning [1,2] have shown promise in this area [3-5], significant challenges remain in ensuring that the data used for model development are representative and reliable. The use case that inspired this work involves a recent project on the dynamic prediction of central line-associated bloodstream infections (CLABSI) using hospital-wide data from the University Hospitals Leuven (Belgium). Multimedia Appendix 1 [6-8] (use case: CLABSI prediction using data from a single-center university hospital) summarizes the main challenges we encountered together with corresponding solutions.

Data collection is part of the routine hospital workflow. Data extraction entails retrieving and structuring raw EHR data from hospital databases using an extract, transform, load (ETL) process, with standards such as Observational Medical Outcomes Partnership Common Data Model (OMOP CDM) facilitating structure and terminology consistency (Multimedia Appendix 1. Glossary of technical terms). Publicly available intensive care unit (ICU) and emergency admission datasets [9-12], such as MIMIC [13,14], support reproducible research, while hospitals also extract private, not publicly shared data for predictive modeling. Data preparation transforms extracted data into a structured format for predictive modeling, often using R (R Foundation) or Python (Python Software Foundation) pipelines. Open-source frameworks, whether generalizable [15,16] or specific to MIMIC [17,18], aim to establish reproducible workflows for EHR data preparation. Data quality issues, such as inconsistencies and artifacts introduced during extraction and preparation, can compromise the performance of predictive models. Understanding the intricacies of data extraction and preparation challenges is essential for developing robust predictive models that can be reliably integrated into clinical practice.

Structured data quality assessments [19-22] are facilitated by frameworks such as by Weiskopf and Weng [23] and METRIC (Measurement Process, Timeliness, Representativeness, Informativeness, Consistency) [24]. However, researchers often conduct unstructured assessments, documenting “lessons learned” from their projects [25-30]. These assessments tend to focus primarily on data cleaning rather than the entire data extraction and preparation process, including cohort definition, outcome definition, and feature engineering. While literature on data quality assessments exists, mitigation strategies remain largely undocumented [31].

## Objective

This paper provides a comprehensive list of challenges encountered during data extraction and preparation using EHR data for developing dynamic prediction models for clinical use. It further proposes recommendations with actionable insights, with the intention to enhance data quality and improve the practical applicability of (dynamic) prediction models in clinical settings. Our insights are drawn from a selective literature review, as well as our experience with various EHR data extractions. This list is intended as a hands-on resource for data extraction engineers (who perform the data extraction) and researchers (who prepare the data for model building) to consult during the extraction and preparation process. By addressing these challenges, we hope to contribute to the development of more reliable and effective predictive modeling frameworks that can ultimately benefit patient care.

We focus on single-hospital structured data, covering both ICU-specific and hospital-wide extractions. We focus on medium to large-scale extractions, that is, generated through structured ETL processes for a large number of extracted items spanning diverse clinical domains (laboratory results, medications, demographics, comorbidities, etc), and we cover standardized (eg, OMOP CMD) and nonstandard, private extractions. We do not address combined data registries, such as national registries (integrating surveys, general practice, insurance data, and EHR extractions), multicenter data extractions, hospital data collected for clinical trials, or the extraction and processing of unstructured data (eg, text notes or images). We cover the broader application of dynamic models (or continuous prediction) for offering the most comprehensive framework, although many of the recommendations are applicable to static prediction models (eg, a single prediction per admission at 24 hours after admission).

Recognizing that EHR software may contain bugs, human errors will occur, and hospital processes will evolve and generally improve over time, we do not provide recommendations for EHR vendors or for modifying hospital workflows, clinical practices, or data recording procedures within EHR systems. When data quality issues, such as those arising from data recording procedures, render the extracted data inadequate for a specific prediction task, we leave it to researchers to assess adequacy, without offering guidance on what constitutes suitable data for a given prediction task.

The section “EHR Data Flow from Data Collection to Model Building” explains the typical trajectory of data, for both model building and clinical implementation, setting the stage and providing context for the detailed list of challenges that follows. The section “Challenges and Recommendations” lists the challenges and actionable recommendations, which represent the core of the paper. These are categorized into 4 groups: cohort definition, outcome definition, feature engineering, and data cleaning. This is one possible categorization, inspired by the stages of the data preparation process. The “Discussion and Conclusion” section summarizes the recommendations and reflects on their broader implications.

## EHR Data Flow From Data Collection to Model Building

**Figure 1:**
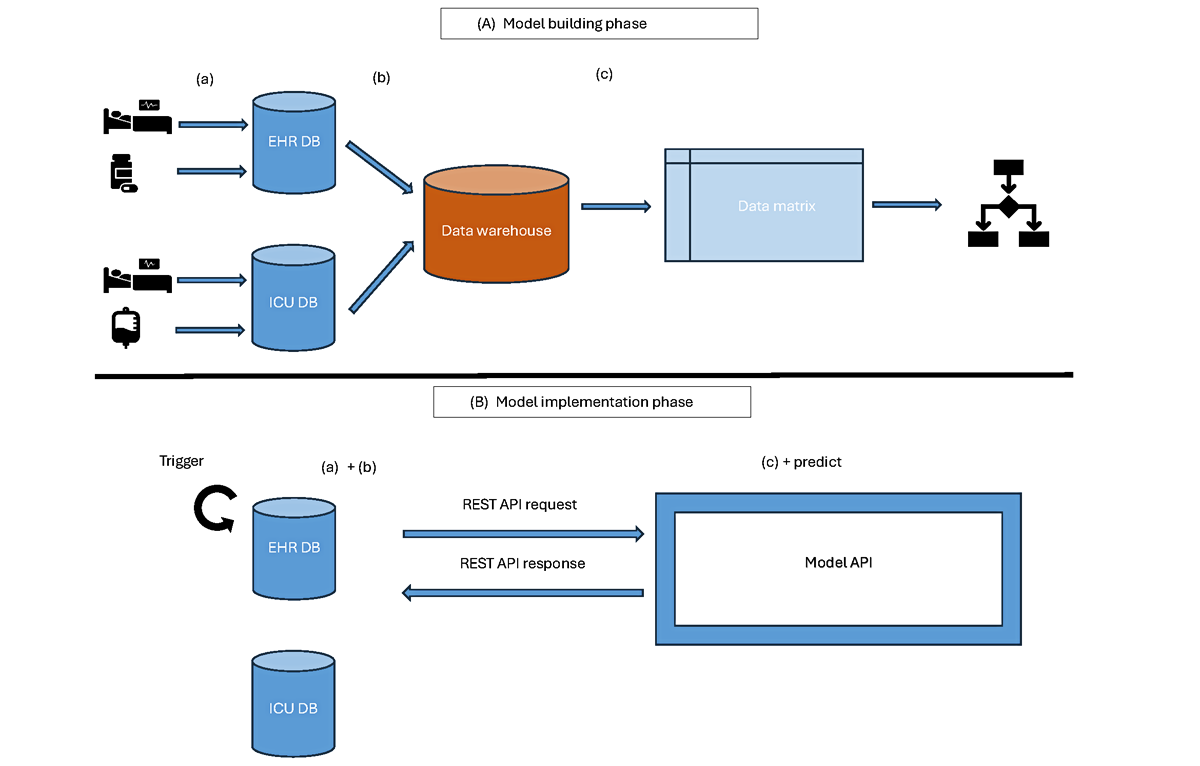
(A) Data flow for the model-building pipeline and (B) model implementation. Two databases are exemplified as data sources, EHR and ICU, although multiple other sources might be used in the hospital’s flow and for data extraction. API: application programming interface; DB: database; EHR: electronic health record; ICU: intensive care unit; REST: Representational State Transfer.

The data flow from the patient’s bed to the prediction model building typically follows three stages: (1) data collection, (2) data extraction, and (3) data preparation (Figure 1A). (1) Patient data are either manually entered in EHR software modules or collected by devices and stored in one or multiple databases. The data collected serve multiple purposes, for example, daily bedside clinical work, national benchmarking [32], or reimbursing the care delivered. (2) EHR data are then extracted from relational databases in a simplified (denormalized) format and typically stored in a data warehouse as multiple “base tables” per category, each capturing different aspects of patient data and health care events. Examples of base tables naming as per OMOP CDM are PERSON, DRUG_EXPOSURE, DEVICE_EXPOSURE, CONDITION_OCCURRENCE, MEASUREMENT, NOTE, OBSERVATION, but naming and granularity of extraction can vary for nonstandard extractions. (3) Researchers building a prediction model either have access to the data warehouse or get a copy of all tables or, under specific ethical and legal considerations, a subset of the base tables or a subset of the patients in the data warehouse (eg, admissions during a specified period of interest for patients undergoing mechanical ventilation). Further, they process the extracted data and bring it in a format on which a prediction model can be built.

At model implementation time in clinical practice in the EHR software (Figure 1B), a trigger (eg, update of laboratory results) or a scheduled task (eg, every 24 hours) will initiate the request for a prediction. Data are already collected (1) for the patient in the EHR databases. The same logic as for extraction (2) is reproduced (typically by reusing the queries or code used for extraction). Using the data packed in a specific format (eg, JSON and XML), the prediction service (typically using a RESTful (Representational State Transfer) application programming interface for communication) is invoked. Data exchange between the EHR and the prediction service is generally performed using the Fast Healthcare Interoperability Resources standard. The prediction service will further prepare the data (3), invoke the model, and return a prediction (and additional information, if foreseen) to the EHR software, which will present it to users in the form of alerts or patient flags within the patient’s chart. The process is typically logged in the EHR system for monitoring purposes. In summary, at real-time prediction, the (1) data collection happens implicitly and is part of the normal clinical flow; (2) data extraction and (3) data preparation are identically reproduced as for model building.

We provide additional background on some particularities for each of the processes of collection, extraction, and preparation, which represent transition phases from a data format to another.

## Data Collection

The EHR database will not reflect with maximum accuracy the “true state” of the patient. First, it will suffer from incompleteness, as not all possible markers and observations can be collected for all patients at all times. The decisions with regards to what data are collected (eg, which laboratory tests are ordered and performed) are highly dependent on the patient’s conditions and on the hospital procedures. Data collected during routine clinical practice are generally documented more carefully when also used for national reporting of quality of care indicators [32]. Sufficient data are collected during clinical practice by doctors, nurses, assistants, etc, in the system to support the patient’s clinical follow-up and treatment. From this perspective, EHR data differ vastly from data collected for clinical trials, where researchers specify the measurements, measurement methods, and collection procedure. Second, nurses and clinicians might have slightly more information than the data collected in the system, either from patient conversations or organizational knowledge. Considering that we do not cover the extraction of text notes and reports, tabular data only will always suffer from a level of incompleteness. Nevertheless, tabular data might prove sufficient for specific prediction tasks. Third, both manual data entry and data collected by devices can be, at times, error-prone, software can have bugs, and data recording procedures in the system will affect the granularity of observed data and will change over time. These considerations, which can be summarized as data completeness, correctness, and currency, as defined by Weiskopf and Weng [23], have to be carefully considered by researchers to assess if EHR data are fit for the prediction goal [21].

## Data Extraction

The extracted data might not correctly reflect the EHR database. Although data extraction generally aims for completeness of all clinically relevant data, the extraction process can introduce undesired artifacts or can have its own limitations. Whenever extraction logic bugs are detected, these should be corrected and safeguarded through unit or integration tests. Mature extraction platforms, tested through repeated use and proven reliability, will generally be less error-prone, and researchers can consider the maturity of the extraction platform as a factor influencing the time they will spend on data preparation. The extraction process is typically carried out by a data extraction engineer, data integration developer, data warehouse engineer, or ETL developer representing the EHR vendor or the hospital information technology department. Hereafter, we refer to this role as the data extraction engineer. They work closely with EHR software developers, hospital information technology staff, and clinical personnel to understand database structures and data recording procedures. They perform clinical concept and terminology mapping and document the extracted data.

## Data Preparation

The prepared data might not correctly reflect the extracted data. Undesired artifacts can be introduced by feature engineering or data cleaning. Good documentation of the extraction format and close collaboration between researchers, data engineers, and clinical experts will ensure that the data and the features are not misinterpreted. Coding errors can always occur. Time-sensitive data poses an additional challenge to ensure no temporal leaks (using future data to predict past events) are introduced by mistake. Outcome leaks (including outcome information in predictors) and test-train leaks (including information from test patients in the training datasets) can also occur if data are not preprocessed carefully. A good practice is to separate the train and test sets first and apply the data preprocessing separately. A modular organization of the code will facilitate unit testing (testing individual functions of a program in isolation to ensure they work as expected) and easy modifications without introducing new errors. The more complex the data preparation, the more error-prone it becomes. The data preparation is performed by a researcher, data scientist, statistician, or machine learning engineer, whom we will refer to as a researcher for the remainder of this paper.

Each stage of processing the data has its own challenges and can introduce new problems, widening the gap between the patient’s state and the data used by the prediction model and ultimately impacting the model building (suboptimal model), model evaluation (misleading performance metrics), or model implementation in clinical practice.

## Challenges and Recommendations

We list common problems originating in the (1) data collection process, the (2) data extraction process, and the (3) data preparation process (Figure 1A). We provide recommendations for mitigation strategies that can be implemented during the (2) data extraction or (3) data preparation. We also focus on problems that can impede the identical reproduction of the extraction and preparation at clinical implementation time. We have categorized the challenges and recommendations into four groups: (1) cohort definition (and inclusion or exclusion criteria), (2) outcome definition, (3) feature engineering, and (4) data cleaning, and each group contains problems originating in the collection, extraction, or preparation process.

We provide mapping of the items to both the Weiskopf and Weng framework [23] and the METRIC framework [24], whenever applicable. Weiskopf and Weng do not include dimensions covering the cohort representativeness and completeness of the extracted features, which are important in prediction settings. We will use the completeness dimension to refer to completeness of data values, as in the original definition [23], as well as completeness of the cohort and of the extracted features (our extension).

While we aimed to make these challenges as generic as possible, we recognize that specific issues are often unique to individual projects and may not apply to all prediction tasks. We advise users to assess the impact of each listed challenge in their specific context. Similarly, the recommendations might not always be universally applicable and depend on the project context. Our guidance remains pragmatic; at times, the best approach may be to “leave as is” to avoid the risk of overcorrection, which can backfire. While certain corrections may improve alignment with clinical meaning for a specific patient group, they can introduce errors or biases when applied to all patients or be difficult to reproduce at clinical implementation time, affecting the model’s performance in clinical use. Following the fitness-for-use principle [20], we encourage readers to remain pragmatic and address only the issues relevant to their data and prediction task.

**Figure 2:**
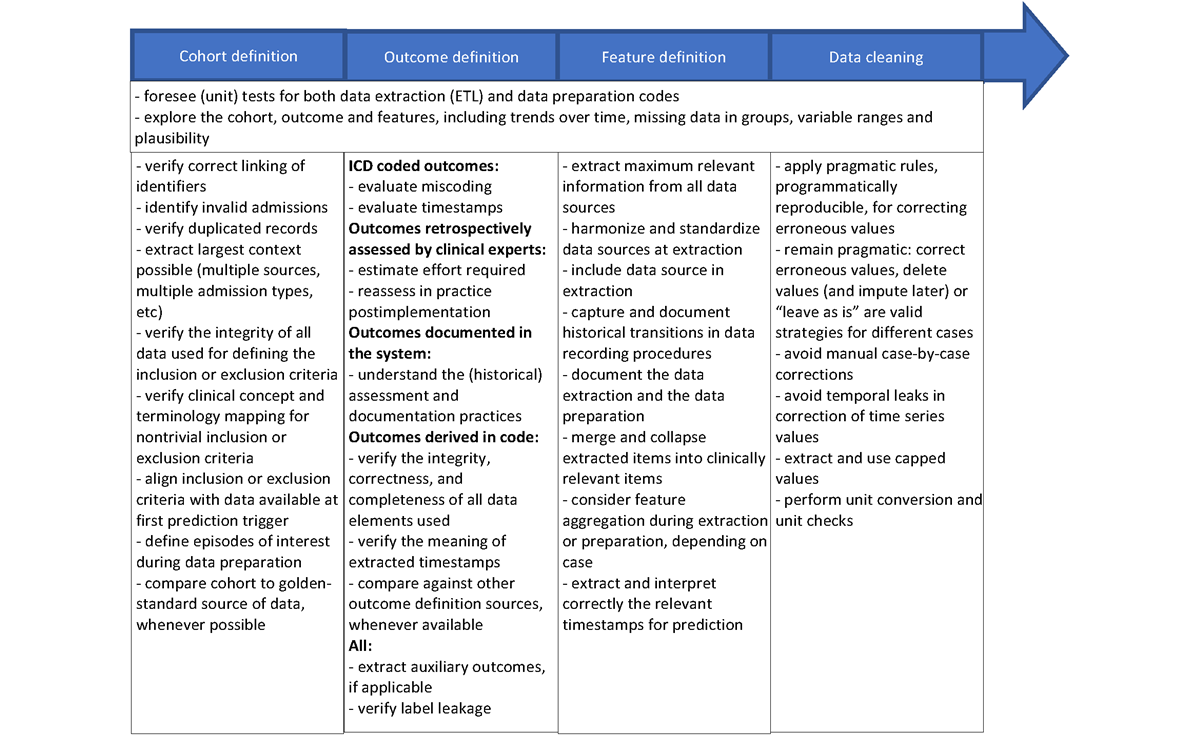
Summary of recommendations. ETL: extract, transform, load; ICD: International Classification of Diseases.

Figure 2 presents a high-level schematic overview of the recommendations, without detailing each specific challenge. For a full understanding of the context in which recommendations are made, we encourage hands-on users involved in data extraction and preparation to consult the following sections and tables below for a comprehensive understanding.

## Cohort Definition

Defining the cohort of interest (eg, hospital-wide population or patients with a specific condition) represents a first step in the prediction task definition (Tables 1-3). It can also be of interest during the project planning phase. An inaccurately defined cohort can lead to selection bias, resulting in performance estimates that do not accurately reflect the model’s performance in clinical practice.

**Table 1:** Cohort recommendations related to identifying admissions.

Problem	Description and recommendation
Linking identifiers	Description: as data sources are relational databases, the extraction process deals with a large number of identifiers. Patient, admission, physician, and even bed [28] identifiers are used for linking clinical items. The process of anonymizing identifiers can also introduce errors. Furthermore, identifiers can differ in different systems (ICU^a^ vs non-ICU). The process of denormalization at data extraction obscures a large number of identifiers. Nevertheless, generally, patient and admission identifiers (anonymized) remain part of the extracted data. Incorrect linking of identifiers can result in an incorrectly extracted cohort. Recommendation: correct linking of obscured identifiers can be tested during data extraction, for example, by verifying if the date of birth of 2 linked patient items from 2 systems match [25]. METRIC^b^: representativeness - depth of data - coverage; WW^c^: completeness
Cross-linking patients and admissions across various data sources creates inconsistencies	Description: patient demographics and admission data are typically stored in a HIS^d^, which acts as a “master” system for patient data and shares these data with other specialized clinical software modules, such as the emergency system, ICU system, and EHR^e^ modules used in other wards. In a flawless integration between systems, all specialized clinical modules maintain identical copies of patient and admission information as the “master” HIS system. However, discrepancies might exist, especially in historical data. Issues such as failed patient merges or demographic updates that do not correctly propagate across all systems can lead to inconsistencies. Additionally, data migration from previous software vendors may introduce discrepancies. For example, a cohort including all patients in the emergency department older than 60 years might be differently identified based on HIS patient demographics than on the demographics from the emergency software. While these situations should normally be rare, they may be more common in historical data. Recommendation: if large discrepancies have been created due to historical reasons, these would ideally be addressed during data extraction, considering the knowledge of historical system integration problems. If discrepancies persist in current clinical practice at the time of model deployment for clinical use, cohort identification and patient demographics may need to reflect the system in which the prediction model is used, for example, if the model is deployed in the emergency department, patient data may need to reflect the information available to emergency physicians. METRIC: representativeness - depth of data – coverage; WW: completeness.
Invalid admissions, not reflecting real patients, are present in the extraction	Description: admissions that do not represent real patients are present in EHR databases [30]. At a minimum, “test patients” on which new EHR software versions are tested will be present in most EHR databases. Other examples can include “ghost” admissions resulting from incorrect data migration from the previous EHR vendor database, or administrative admissions for beds or equipment to enable laboratory sample collection. Such invalid admissions might or might not be linked to a patient record. Such admission can occur only during the training data timeframe (for historical reasons, such as data migration problems), or both at training and clinical implementation time (for test patients). Recommendation: test patients can be easily filtered during data extraction by excluding a list of known test patients or admission identifiers; these can be difficult to detect during data preparation. Detecting invalid admissions due to historical problems requires knowledge of the database and the historical hospital workflows; generally, these are best handled during data extraction. If invalid admissions are detected during data exploration, the root cause of the underlying problem should better be discussed with data extraction engineers. Inclusion of such admissions at clinical implementation would generally not represent a real danger, because health care professionals are aware that predictions created for admissions that do not represent a real patient can be safely disregarded, and in most workflows, these would not interrupt their workflow. The impact on clinical implementation should, though, be evaluated on a case-by-case basis. METRIC: informativeness – redundancy - uniqueness; WW: not applicable.
Duplicated admissions are present in the extraction	Description: the same patient admission is extracted twice, with different identifiers. This can occur due to extraction in separate batches or identifiers anonymization, and leads to a biased cohort definition. Duplicated admissions can also be recorded in the system for administrative or funding purposes. Recommendation: such problems can be captured in the data extraction framework using unit or integration tests. During data preparation, tables can be checked for duplicate records. METRIC: informativeness - redundancy - uniqueness; WW: not applicable.
Restricting extraction to hospitalized patients may lead to the omission of useful patient data	Description: often, data extraction is restricted to hospitalized patients (also called inpatient admissions), excluding outpatient (consultations) or day-care admissions. Some hospitalized patients may have relevant data recorded during previous outpatient admissions. Depending on hospital workflows, certain prediction tasks, such as those for planned surgeries, may benefit from preadmission data recorded during outpatient visits. For instance, laboratory samples ordered 1 or 2 days before an inpatient admission may be linked to an outpatient visit and could be clinically relevant for the prediction task. Recommendation: extraction can be extended to all hospital admissions (inpatient, outpatient, and day-care), and researchers can decide if outpatient or day-care admissions contain useful data for the prediction task. METRIC: representativeness - depth of data - coverage; WW: completeness.

^a^ICU: intensive care unit.

^b^METRIC: Measurement Process, Timeliness, Representativeness, Informativeness, Consistency Cluster and Dimension Mapping.

^c^WW: Weiskopf and Weng dimension mapping.

^d^HIS: hospital information system.

^e^EHR: electronic health record.

**Table 2:** Cohort recommendations related to inclusion and exclusion criteria.

Problem	Description and recommendation
Incorrect or incomplete mappings leading to an incorrect cohort definition applied at extraction	Description: for some prediction tasks, only a subset of hospitalized patients is of interest, for example, patients with a central catheter or urinary catheter, patients undergoing surgery (for postoperative outcomes), patients on mechanical ventilation, or patients with a specific diagnosis or condition. The items that identify a device (a mechanical ventilator or a catheter) or the definitions used for defining a patient condition might differ between medical wards. Correct identification of these items during data extraction might be challenging, and inadvertently, items may be missed, creating either gaps in data or incorrectly excluding admissions. Often, it is difficult to spot such incorrect exclusions during data preparation, as the researcher does not have access to or knowledge of the EHR system configuration, hospital workflow, and data recording procedures in each department. Incorrect exclusion can be due to many factors, such as typos (an item has been configured with a typo for some period in the system, eg, central vs central venous catheter) or different recording procedures in specialized wards (eg, dialysis catheters are recorded in a special form in the nephrology department), ultimately leading to sample selection bias [26]. For identifying patient conditions, multiple sources can be used, such as ICD^a^ codes or discharge diagnoses. Recommendation: if ethical approval can be granted to extract all admissions within the timeframe of interest, without applying other specific exclusion criteria, the cohort definition can be carried out during data preparation, as a collaborative effort between data extraction engineers, researchers, and clinical representatives. This allows researchers to perform additional verification, improving the cohort definition through multiple reviews. Whenever this is not possible, the data extraction engineers would have to work closely with clinical representatives for the correct cohort definition. Correct clinical concept and terminology mapping becomes crucial. METRIC^b^: representativeness - depth of data - coverage; WW^c^: completeness
Inclusion or exclusion criteria require patient history before the study period	Description: inclusion or exclusion criteria based on a history of chronic conditions, such as osteoarthritis [21], may not be fully recorded in the EHR system or might require extracting data from a period preceding the study timeframe, for example, a “look-back period” of 3 to 5 years, as historical conditions are associated with previous admissions. Recommendation: assessing the completeness of data used to define the inclusion or exclusion criteria before study initiation can be done through an external source linking to gold standard data sources [21]. Alternatively, if historical diagnoses relevant to the current admission are available in (structured or unstructured) clinical notes recorded shortly after admission, these can be used. METRIC: representativeness - depth of data - coverage; WW: completeness.
Restrictive inclusion or exclusion criteria applied at the extraction limit the scope of missing data imputation	Description: imputation of missing values (eg, laboratory results) might benefit from the extraction of a larger cohort than the one strictly of interest. Similarly, if the admissions are further split in episodes of interest for prediction, which are segments of time during patient admissions when a patient is considered at risk for the outcome of interest (eg, catheter episodes or mechanical ventilator episodes), the imputation can be carried out in the largest possible context (using all patient-days during an admission instead of only the episode-patient days). Performing missing data imputation in a larger context would, in theory, be beneficial, especially if the cohort of interest is rather small, although there is no evidence of this topic being studied. Recommendation: if ethical approval can be granted to extract all hospitalizations during a specified time frame, a cohort definition using inclusion or exclusion criteria can be carried out during data preparation, eventually after missing data imputation. METRIC: representativeness - depth of data - coverage; WW: completeness.

^a^ICD: International Classification of Diseases.

^b^METRIC: Measurement Process, Timeliness, Representativeness, Informativeness, Consistency Cluster and Dimension Mapping.

^c^WW: Weiskopf and Weng dimension mapping.

**Table 3:** Cohort recommendations related to episodes of interest for prediction.

Problem	Description and recommendation
Discontinuity or fragmentation of the patient stay due to cross-linking across different data sources	Description: whenever the cohort definition is carried out at extraction, for example, admissions for patients with a specific device, and data are extracted from multiple databases (ICU^a^ and hospital-wide EHR^b^), data for the entire hospital admission have to be extracted from both systems. For example, a patient admitted through the emergency, transferred to the ICU, and then to the nephrology ward has a dialysis catheter only during the ICU stay. Extracting only the data during the ICU stay might not be sufficient for the prediction task. Data before ICU contains predictive information. Often, for devices such as catheters, the patient is considered at risk of catheter-associated infections 48 hours post catheter removal; therefore, the patient is still at risk for the event of interest for prediction post-ICU stay, during the stay at the nephrology ward. Alternatively, discontinuities can happen for extracted data when a patient is transferred between wards due to overlooked mapping of clinical items from more systems (eg, a patient has a central catheter in the general ward, is transferred to the ICU without the presence of the central catheter, and gets transferred back to the general ward, with reappearance of the central catheter). Recommendation: whenever the hospital admission data is located in multiple data sources (ICU and EHR), data relating to the entire hospital admission should be extracted, even if the inclusion criteria are met only in one of the data sources. METRIC^c^: representativeness - depth of data - coverage; WW^d^: completeness
Temporal leaks in cohort definition	Description: a subgroup of patients with a specific condition might be of interest for the prediction task, for example, cardiac patients, but predictions are made during the entire hospital admission. Defining the subgroup using criteria that are not available at the start of admission (or at the first prediction trigger), such as discharge ICD^e^ codes (coded typically after patient discharge), or patient presence in the cardiology ward (while the patient was admitted through emergency, was transferred to the ICU and then to the cardiology ward), will affect the applicability of the model. Recommendation: the cohort should be defined using data available at the first prediction trigger, for example, data present at admission (eg, admission reason) or data available during previous admissions (eg, patients with ICD codes indicative of cardiovascular conditions during previous admissions). Alternatively, the first prediction trigger can be defined when data becomes available: predictions start at patient transfer to the cardiology department and end at patient discharge. Inclusion or exclusion criteria should never be defined using data after the prediction trigger, for example, excluding patients who died for predictions during the patient’s hospitalization. METRIC: timeliness - currency; WW: currency.
Definition of episodes of interest	Description: the patient follow-up time relevant for the prediction task might be different from the admission timeframe, from admission time until discharge time. This can either mean fragmenting hospital admissions in episodes of interest: catheter episode, mechanical ventilation episode, postoperative follow-up, or, conversely, merging subsequent overlapping hospital admissions [20,30]. Defining the episodes of interest at data extraction time by using date filtering in large queries can prove to be error-prone. Recommendation: defining the episodes of interest during data preparation, rather than during data extraction, might prove less error-prone and comes with the advantage of benefiting from a larger patient context for prediction. Extraction of full admissions is recommended. METRIC: representativeness - depth of data - coverage; WW: completeness.
Episode fragmentation	Description: definition of episodes of interest, such as catheter episode and mechanical ventilator episodes, can be carried out by start and end times: catheter placement and catheter removal, start and end time of mechanical ventilator, whenever these are available in the system and recorded at their time of occurrence. We warn that using the coded procedures (CPT^f^ codes) start and end times might hinder the model’s applicability, as these are generally used for billing and registered in the system post-factum, sometimes at the end of the admission. Whenever start and end times are not available for prediction time, the registration of specific observations (eg, catheter monitoring observations) or parameters (PEEP^g^ and FiO2^h^ for mechanical ventilation) identifies an episode. These observations or parameters can exhibit gaps in registration. Observations can be recorded manually in the EHR system (catheters), or recorded by devices and integrated in the EHR system with a prespecified granularity, as hourly or every minute (mechanical ventilation). Whenever recorded manually, they can display weekend effects, that is, fewer or no values recorded during weekends due to understaffing, leading to data gaps. Whenever recorded by devices, the fragmentation might depend on the granularity of measurements integrated, which can be set differently per monitoring device. Recommendation: exploration of the episodes can be done during data preparation, by checking the number of episodes per patient, the episode length, and the time between episodes. Comparison to procedure-coded placement and removal time can be carried out whenever these are available, but we discourage using procedure codes if these are not registered in the system in real time. METRIC: measurement process - completeness; WW: completeness.

^a^ICU: intensive care unit.

^b^EHR: electronic health record.

^c^METRIC: Measurement Process, Timeliness, Representativeness, Informativeness, Consistency Cluster and Dimension Mapping.

^d^WW: Weiskopf and Weng dimension mapping.

^e^ICD: International Classification of Diseases.

^f^CPT: Current Procedural Terminology.

^g^PEEP: positive end-expiratory pressure.

^h^FiO2: fraction of inspired oxygen.

## Outcome Definition

Prediction models using EHR data usually focus on in-hospital or postdischarge outcomes, including mortality, length of stay, readmission, acute events (such as bacteremia, sepsis, and acute kidney injury), and chronic diseases (such as heart failure, cancer, and cardiovascular disease) [33]. We focus on outcomes that can be derived solely from structured EHR data, without linkage to external data sources or extraction of text notes, and are linked to a patient; that is, we exclude resource use and workflow optimization outcomes (Tables 4 and 5). Similar to the cohort definition, a good practice is assessing the feasibility of defining the outcome during the project planning phase.

**Table 4:** Outcome recommendations for outcomes directly available in the extraction.

Problem	Description and recommendation
Outcomes derived based on *ICD*^a^ codes are not timestamped and often unreliable (under- or upcoded)	Description: diagnoses are typically recorded by coders using the ICD-9^b^ or ICD-10^c^ coding system for reimbursement purposes, typically after patient discharge and without reflecting the time when the diagnosis was established. In some cases, though, these could be coded during the patient’s admission, although these situations are rare. Coded diagnoses are subject to undercoding, upcoding, or creep (misspecification or miscoding) [34] or can represent the diagnostic differential (other possible conditions considered before confirming a final diagnosis) rather than the actual diagnosis [27,35]. Coding practices can vary based on admission types; for example, ICD codes for emergency patients may be restricted only to the presenting complaint, while ICD codes for inpatients may reflect chronic diseases and comorbidities unrelated to the presenting complaint. Typically, there will be more than one code that clinically identifies a disease (eg, chronic kidney disease); clinical knowledge is needed to map codes to the outcome label. Historical migration, eg, from ICD-9 to ICD-10, might have introduced discontinuity or changes in coding practice [34]. ICD codes might evolve over time, for example, the introduction of a code for COVID-19 in 2020. Due to a lack of timestamps, ICD codes are generally inappropriate for the prediction of acute events during a hospital admission. Differences in coding practices can introduce bias in the outcome defined by the ICD codes. Recommendation: understand the hospital’s coding practices, including potential under- or upcoding, and ensure that relevant timestamps for the event can be established. Explore temporal trends, as the coding practice might have changed over time. METRIC^d^: measurement process - source credibility - traceability; timeliness - currency; WW^e^: currency
Outcomes retrospectively assessed by clinical experts are labor-intensive and subject to human error	Description: clinical experts can determine outcomes by reviewing patient records retrospectively, either systematically at regular intervals (eg, an infection preventionist reviewing records monthly to report hospital-acquired infections to national public health institutes) or specifically for a prediction task (eg, labeling patient records in extracted data). Both approaches require significant time and effort and are prone to errors. Recommendation: one way to reduce human error is to involve a group of experts for consensus-based outcome assessment. However, this makes the task even more labor-intensive. Additionally, research or hospital teams may want to evaluate the model performance after clinical implementation, for example, 1 year post deployment. In this case, outcomes will need to be assessed by the same experts using the same procedure as during model training, which is rarely feasible. METRIC: measurement process - human-introduced error - noisy labels; measurement process - completeness; WW: correctness.
Outcomes documented in the system are subject to mislabeling and delay in registration	Description: assessments carried out during the patient’s stay, for example, delirium assessment by a neurologist using CAM^f^ or DRS^g^, can be used to define the outcome. These can be subject to different documentation practices in different wards or patient groups. For example, delirium might be consistently evaluated after surgery, but inconsistently in patients who did not undergo surgery. There may also be delays in documentation compared to the clinical assessment time (eg, documented at the end of the day), which can impact time-sensitive outcomes. Recommendation: understanding the assessment practices of the events and identifying the outcome is essential in defining their reliability. Understanding the documentation practices (and the registration delay compared to the actual event time) can also be important, depending on how time-sensitive the predictions are, how often the predictions are renewed, and the prediction time horizon. This information is typically retrieved from clinical experts, as documenting every single clinical assessment practice during data extraction would be overwhelming. Manual relabeling by clinical experts post extraction, based on patient assessments, possibly combined with other data such as clinical notes, can prove to be a viable solution for some outcomes, although it remains a labor-intensive task. METRIC: informativeness - understandability; measurement process - human-introduced error - noisy labels; measurement process - completeness; timeliness - currency; WW: correctness; currency.
Specific prediction tasks require the extraction of auxiliary outcomes or competing events	Description: deep neural network models may benefit from the use of auxiliary outcomes (eg, predicting the levels of creatinine, urea nitrogen, sodium, potassium, chloride, calcium, and phosphate, along with AKI^h^) as a form of regularization [3,4]. Competing events (such as death, discharge, or device removal) are sometimes modeled when using regression models [6,21]. Recommendation: check the availability of auxiliary outcomes or competing events. METRIC: informativeness - feature importance; WW: completeness.
Label leakage due to temporal leakage	Description: whenever the prediction time is too close to the event time, strong features will rather detect than predict the outcome that is already known to the health care providers. Examples are mortality prediction at the disconnection of the mechanical ventilator as a family decision to withdraw care [35], and including time points after the occurrence of the event of interest in model training and evaluation [36]. Recommendation: carefully define the outcome label. For example, contact with palliative care, transfer to palliative care, or death can be used as an outcome definition for mortality prediction. Ensure post event data is excluded during data preparation. METRIC: not applicable; WW: not applicable.

^a^ICD: International Classification of Diseases.

^b^ICD-9: International Classification of Diseases, Ninth Revision.

^c^ICD-10: International Statistical Classification of Diseases, Tenth Revision.

^d^METRIC: Measurement Process, Timeliness, Representativeness, Informativeness, Consistency Cluster and Dimension Mapping.

^e^WW: Weiskopf and Weng dimension mapping.

^f^CAM: Confusion Assessment Method.

^g^DRS: Delirium Rating Scale.

^h^AKI: acute kidney injury.

**Table 5:** Outcome recommendations for outcomes derived using clinical or surveillance definitions.

Problem	Description and recommendation
Outcomes derived using clinical or surveillance definitions often require the extraction of multiple data sources	Description: clinical or surveillance definitions provide a more robust alternative compared to ICD^a^ codes or documentation of clinical assessments for certain acute events (eg, AKI^b^, CLABSI^c^, and VAE^d^). It comes with advantages: the event occurrence or onset timestamp can be established, and the same definition is used over the entire training and test data, but its consistency might still depend on the recording practices of the items used in the definition. Furthermore, these items span across multiple data sources, for example, for CLABSI, admission data, patient age data (in months, with a focus on neonates), laboratory, microbiology, vital signs, and imaging reports are necessary to fully comply with the definition. Recommendation: all data sources and their completeness should be checked to decide the appropriateness of the extracted data for outcome derivation. This is typically carried out during data preparation. Additionally, we recommend extracting both the clinically relevant timestamp as well as the timestamp when data are recorded in the system, whenever these differ (examples in Table 8), and using the clinically relevant timestamp for outcome derivation, for example, the sample collection time for creatinine when calculating AKI outcome. METRIC^e^: measurement process - completeness, representativeness - depth of data - granularity; WW^f^: completeness
Clinical or surveillance definitions are complex, and their implementation is prone to variations	Description: the definition logic for clinical or surveillance definitions is documented in large documents, typically issued by health authorities such as CDC^g^, and the logic is generally complex. As these are replicated in code during data preparation using their documentation as the coding requirements, a good understanding of the definition is necessary. For example, the VAE definition is time-sensitive, and there are multiple corner cases (parts of the definition implementation that require a specific adapted logic). The definitions can be subject to interpretation, and there will be variation if different people implement them based on the same documentation. Moreover, coding bugs can occur when implementing complex logic. Recommendation: unit tests can be foreseen in data preparation for corner cases and time-sensitive interpretation. A good understanding of the definition and its clinical interpretation can be achieved through communication with clinical experts. Code reviews can also capture misinterpretations. Whenever other definitions are historically available (eg, ICD codes, CLABSI definition manually assessed and documented by infection preventionists), these can be used for detecting deviations. Some detected deviations might help with correcting programming errors, but not all deviations will necessarily constitute a problem. METRIC: not applicable; WW: not applicable.
Outcomes derived using clinical or surveillance definitions can be affected by missing or erroneous data	Description: the retrospective calculation of clinical or surveillance definition relies on the availability of different items in the system, which in turn relies on the hospital process of recording these items. For example, CLABSI or VAE events include fever as a symptom check, which in turn, can be derived by using temperature values. Temperature values can be missing, and errors in recording these values will be present. Recommendation: the data recording frequency and its impact on the outcome calculation should be assessed. The necessity of cleaning or inputting the data before outcome calculation should be assessed. Alternatively, assumptions can be made (eg, microbiology results are always ordered in the presence of fever) to simplify the definition, but such assumptions should be carefully reviewed with clinical experts. METRIC: measurement process - completeness; representativeness - depth of data - granularity; WW: completeness.

^a^ICD: International Classification of Diseases.

^b^AKI: acute kidney injury.

^c^CLABSI: central line-associated bloodstream infection.

^d^VAE: ventilator-associated event.

^e^METRIC: Measurement Process, Timeliness, Representativeness, Informativeness, Consistency Cluster and Dimension Mapping.

^f^WW: Weiskopf and Weng dimension mapping.

^g^CDC: Centers for Disease Control and Prevention.

## Feature Engineering

Feature engineering is the process of selecting, transforming, and creating features (variables) from the extracted EHR data to ensure that the data are brought into a suitable format for modeling and that relevant information is processed in a meaningful way. It includes data mapping (eg, mapping medication brand names to generic drug names) and transformation (eg, grouping medical specialties in meaningful categories), converting timestamped events (eg, laboratory results, medication administration, and vital signs) into snapshot-based features, and data aggregation (summarizing multiple observations or measurements into single features). The feature engineering is typically performed during data preparation, and usually it happens concomitantly with data cleaning. Sometimes feature engineering is also performed at data extraction time, for example, for reducing the data volume for high-frequency time-series data, especially in ICU settings, or for computing scores that are calculated and displayed in the EHR software but not stored in the system. Clinical concept mapping, such as mapping medication to Anatomical Therapeutic Chemical codes, laboratory tests to LOINC (Logical Observation Identifiers, Names, and Codes) codes or procedures and clinical observations to Systematized Nomenclature of Medicine Clinical Terms can occur at different stages, including within the EHR software, during data extraction (eg, OMOP CMD Standardized Vocabularies), or as part of data preparation.

We distinguish between generic feature engineering and time-sensitive feature engineering. Generic feature engineering (Tables 6 and 7) deals with the mapping of clinical items Time-sensitive feature engineering (Tables 8 and 9), which deals with data aggregation for which correct processing of timestamps is critical and that can result in temporal leaks (ie, data available at a specific time for training the model, but not available in the system at that timestamp. Time-sensitive feature engineering is critical for dynamic prediction models, can also be useful for some static models (eg, predictions at 24 hours after admission), and has less impact on models with a prediction trigger at the end of the admission (eg, readmission prediction). We acknowledge that some minimal temporal leaks might not be very detrimental for the model performance or for its applicability, while others can have a large impact. However, following the “do it right the first time” principle, a good understanding of the extracted timestamps and correct handling of dates or times during data extraction and preparation can safeguard against future problems, big or small.

As for the previous 2 groups, certain aspects of feature engineering can be evaluated during project planning, at least for the key features relevant to the prediction task and, at a minimum, for their availability. For instance, if a key feature was only recorded for a limited period before being discontinued or if timestamps of key features are unavailable for a dynamic prediction task, the project may become jeopardized.

**Table 6:** Feature engineering recommendations (not time-sensitive) related to clinical concept mapping.

Problem	Description and recommendation
Inconsistencies due to the data extraction of the same clinical items from various databases	Description: there will be a variation in the data extraction procedure when data sources differ. A typical example is extractions from EHR^a^ (hospital-wide) and ICU^b^ systems. These often follow different structures (eg, units, such as centimeters for neonates length or meters for adults’ height, are available or units are implicitly considered the same unit; devices registered with start and end time in ICU, and through a “device present” in the EHR), have different meaning (eg, different pain scales used in different age groups), can be recorded only in one system, or recorded in a structured way in 1 system and in free text in another. Recommendation: one approach is to extract the items in a harmonized format. Alternatively, extract separate base tables for each data source. The maximum possible information should be extracted from all systems during data preparation. If data sources are harmonized at extraction, include the data source, as cleaning steps might differ (eg, cleaning temperatures recorded by devices in the ICU can be different than cleaning errors in manually entered temperatures in other wards). Standardization at extraction using OMOP CMD^c^ helps in harmonizing the data structure and the clinical concepts terminology across data sources. METRIC^d^: consistency - rule-based consistency - syntactic consistency; WW^e^: not applicable.
The same clinical concept is available in different tables in the same database	Description: differences in registration can occur in the same database. The registration of specific clinical concepts can deviate from the standard procedure in specialized wards. For example, catheter information may be recorded in a standard structured way for all wards (using nursing observations), but dialysis catheters are recorded in a specialized software module in the nephrology department. The same can happen with specialized software modules deployed over time, and items being recorded in a database table in older data and in another table in newer data. Additionally, some fields might represent patient-reported data while others represent clinically assessed data [27], and depending on the research question, either both or only the clinically assessed data can be relevant. Recommendation: capture recording practices and historical transitions during the clinical concept mapping phase at extraction, for each underlying table. Data exploration during data preparation should reveal only changes in hospital processes if the mapping has been correctly performed during extraction. METRIC: consistency - rule-based consistency - syntactic consistency; WW: not applicable.
Clinical concept mapping differs between wards or over time	Description: even when the same clinical concept is recorded in the same table, configuration catalogs might have changed over time or might differ in different wards. Configuration changes (eg, CVC^f^ vs central venous catheter) or even typos can create gaps in data if not mapped correctly. Recommendation: as a step in the extraction phase of the ETL^g^ process, perform clinical concept mapping in collaboration with information technology or clinical experts who know the historical use of the EHR software in the hospital, including changes in configuration catalogs. OMOP CDM provides standardized mapping, but does not solve the problem of correctly detecting all original items to be mapped. METRIC: measurement process - human-introduced error - carelessness; consistency - rule-based consistency - syntactic consistency; WW: correctness.
Partially overlapping data regarding the same clinical concept is available in multiple extracted tables	Description: often, multiple extracted tables contain the same clinical information. For example, diagnoses might be coded using ICD^h^ codes at the end of the admission, but they might also be available from care pathways recorded during the admission. Alternatively, ICD-9^i^ and ICD-10^j^ coding might coexist [20]. Medical procedures are coded using CPT^k^ codes, typically after discharge, but information on the performed procedures might be available in structured fields in the EHR during the patient’s admission. Medication orders, prescriptions, and administration might represent 3 different extracted tables. Recommendation: provided clear extraction documentation, the decision either merging extracted items into 1 feature or making a choice of a source against the other is generally taken during data preparation. Data source agreement exploration can be carried out during data preparation. METRIC: informativeness - redundancy - conciseness; consistency - rule-based consistency - compliance; documentation; WW: concordance.
Changes in hospital processes over time result in gaps in the extracted features	Description: gaps in recording features over large periods (months or years) can occur. The EHR software or specific software modules are generally deployed ward by ward, and such gaps in data can be seen in the first years of the extracted period, before hospital-wide adoption of the software (eg, if an emergency department started using a module in 2014 but data extraction starts in 2012). Gaps can also occur during transition periods from 1 coding system to another (eg, from ICD-9 to ICD-10 as the hospital is not legally obliged to code the diagnosis codes for 1 calendar year). Software bugs occurring for a limited period can also create data gaps, as can (temporary) discontinuation of specific recordings, for example, to ease the nurses’ workload. Recommendation: document changes whenever known at extraction. This warns the researcher of the usefulness of extracted features for the prediction task. Explore trends over time in the recording of each feature (hospital-wide and per ward) to detect if gaps are present. Possible decisions are to not use unreliable features, proceed with missing data imputation, or redefine the period of interest or the cohort of interest. METRIC: measurement process - completeness; documentation; WW: completeness.

^a^EHR: electronic health record.

^b^ICU: intensive care unit.

^c^OMOP CDM: Observational Medical Outcomes Partnership Common Data Model.

^d^METRIC: Measurement Process, Timeliness, Representativeness, Informativeness, Consistency Cluster and Dimension Mapping.

^e^WW: Weiskopf and Weng dimension mapping.

^f^CVC: central venous catheter.

^g^ETL: extract, transform, load.

^h^ICD: International Classification of Diseases.

^i^ICD-9: International Classification of Diseases, Ninth Revision.

^j^ICD-10: International Statistical Classification of Diseases, Tenth Revision.

^k^CPT: Current Procedural Terminology.

**Table 7:** Feature engineering recommendations (not time-sensitive) related to feature availability and aggregation.

Problem	Description and recommendation
Ward-specific data recording patterns	Description: the difference between wards will exist in compliance with registration of specific care items and adherence to registration guidelines. These differences may be due to staffing requirements or may be due to perceived clinical relevance for their specific patient population. Specific scores and scales may apply only to specific patient groups. Missingness patterns of extracted items may therefore differ between wards. Recommendation: during data preparation, missing data patterns can be explored in each ward and appropriate decisions can be taken depending on context: either impute missing data or engineer features in a contextually relevant manner, for example, using GCS^a^ scores only in appropriate patient groups. METRIC^b^: measurement process - completeness; WW^c^: completeness.
Weekend effects result in missing data	Description: due to staffing constraints, certain clinical items (eg, registered by nurses) can be recorded with a lower frequency during weekends or public holidays. Recommendation: exploring the extracted items or features trends over days of the week can help with detecting such trends. During data preparation, creation of separate features for the days of the week (Monday to Sunday) or weekend flags, eventually together with interactions with the features that display weekend gaps, can help the model distinguish between important patterns during weekdays versus weekend days. METRIC: measurement process - completeness; WW: completeness.
The feature set definition is too restrictive	Description: depending on the project approach, researchers might have to define the set of clinical items to extract at the start of the project. Typically, the important predictors for the outcome of interest are selected based on a literature review or input from clinical experts. It can, though, prove interesting to model the prediction task using a competing events framework [6,7]. Auxiliary outcomes are often used as a form of regularization in deep learning applications [3]. In these situations, extracting items with predictive value for the competing events or auxiliary tasks can prove useful. Additionally, missing data imputation of extracted features can benefit from the extraction of more features that have value in imputing the features of interest in the prediction task. Recommendation: whenever possible, the extraction of a larger set of clinical items of interest can benefit the data preparation process. METRIC: informativeness - feature importance; WW: completeness.
Overmapping during extraction can dilute the predictive signal	Description: although terminology mapping during data extraction can significantly reduce the time spent during data preparation, mapping into too broad categories can dilute the predictive signal. For example, ICD^d^ to CCS^e^ or CCSR^f^ mapping might be performed at extraction. Depending on the outcome to be predicted, the history of specific ICD codes might prove more predictive than broader categories. Recommendation: both the more granular extraction as well as the broader mapping could be made available during extraction, for example, both ICD and CCS codes. METRIC: informativeness - feature importance; WW: concordance.
Use of hospital aggregate features	Description: the prediction task might benefit from the use of aggregate ward-level features, such as bed occupancy or nurse or clinician workload at the prediction time, typically aggregated per calendar day. Such features can, in principle, be calculated during data preparation based on the extracted data. At the prediction time in clinical implementation, though, the prediction API^g^ should receive all data for all patients hospitalized at prediction time for the calculation of patient census or bed occupancy. This constitutes an unnecessarily heavy data transfer for making predictions for 1 patient at 1 point in time. Moreover, if the inclusion or exclusion criteria are applied at extraction (eg, patients on mechanical ventilation), the researcher does not have access to the precise number of patients that occupy beds in the ward, as not all patients are on mechanical ventilation. Recommendation: it is more convenient to foresee such aggregate features during data extraction, considering that the same logic used at extraction can be reused at the clinical implementation prediction time. Reusing the same database queries at prediction time reduces the need for large data transfers and exposure of all hospitalized patient data to the prediction API. METRIC: informativeness - feature importance; measurement process - completeness; WW: completeness.
Use of patient aggregate features	Description: baseline features capturing aggregates of previous patient history, for example, historical comorbidities often prove useful for the prediction task. These can be calculated either during data extraction or at prediction time. Calculating these during data preparation comes with downsides. First, the extraction might be limited to a specific time range, for example, admissions from 2014 to 2020. Patients admitted in 2014 will be subject to left censoring for historical comorbidities, as their admissions before 2014 are not included in the extracted data. Second, at clinical implementation time, data for all patient admissions from the past would have to be sent to the prediction API, which might result in unnecessarily large data transfers over the network, especially if predictions are renewed often for a large number of patients. Last, when the exclusion or inclusion criteria are defined during data extraction, not all prior admissions for a patient are available (a patient undergoing surgery during the current admission might have had prior admissions without surgery). Recommendation: preferably, these would be calculated during data extraction and identically reproduced in clinical implementation. Within certain limitations, these can also be derived at preparation time. METRIC: informativeness - feature importance, and measurement process - and completeness; WW: completeness.

^a^GCS: Glasgow Coma Scale.

^b^METRIC: Measurement Process, Timeliness, Representativeness, Informativeness, Consistency Cluster and Dimension Mapping.

^c^WW: Weiskopf and Weng dimension mapping.

^d^ICD: International Classification of Diseases.

^e^CCS: Clinical Classifications Software

^f^CCSR: Clinical Classifications Software Refined

^g^API: application programming interface.

**Table 8:** Feature engineering recommendations (time-sensitive) related to timestamp integrity.

Problem	Description and recommendation
Timestamps shifting for anonymization	Description: as part of the anonymization process applied at data extraction, date shifting can be applied to deidentify the date of patient admissions [13,14,28]. This process should preserve the sequence of admissions of a patient, the sequence of events within an admission, as well as seasonal features (eg, time of day, day of the week, and month of the year). However, the process can introduce artifacts and render specific feature extractions during data preparation impossible (eg, bed occupancy and nurse workload). Recommendation: the anonymization process applied at extraction should be tested using unit or integration tests in the data extraction framework. Whenever this process renders the extraction of certain features unfeasible during data preparation, this should be documented. METRIC^a^: timeliness - currency; documentation; WW^b^: currency.
Timestamps when data are available in the system for real-time prediction differ from the clinically relevant timestamps	Description: the clinically relevant timestamp reflects the time that best corresponds to the patient’s actual physiological state, and can differ significantly from the data recording timestamp. For laboratory (and microbiology) results [3,26], the relevant timestamps in the clinical workflow are order time (when the clinician orders a laboratory test), planned collection time, collection execution time (when the nurse collects the sample), preliminary result availability time, and final (validated) result availability time. Typically, the sample collection time and final result time are extracted from the system. For example, the time when a positive microbiology result is available in the laboratory system is often more than one day after the blood collection by the nurse; the latter is the clinically relevant timestamp because the result reflects the patient’s state at blood collection time (1 day earlier than the result availability). Using the wrong timestamp in prediction will create temporal leaks, but its impact on the individual predictions will differ depending on how features are aggregated in time windows and how often the predictions are renewed (eg, hourly, daily, or trigger-based when new data become available). Ward transfers can also be entered in the system before or after the actual patient care starts [30]. Recommendation: most of the time (and especially for laboratory results), the relevant timestamps can be extracted from the system. If not, the extraction documentation should mention the meaning of the extracted timestamp, its usual recording procedure, and possible delays in recording. To avoid time leaks at implementation time, the registration time in the system (result time for laboratory results) should be used for features included in the prediction model. On the contrary, for outcomes calculated retrospectively based on extracted laboratory results (eg, AKI^c^, sepsis, and CLABSI^d^), the clinically relevant timestamp (sample collection time for laboratory results) should be used for outcome calculation, as mentioned in Table 2. METRIC: timeliness - currency; documentation; WW: currency.
Extracted timestamps may reflect the time of the last modification rather than the original creation time of an item	Description: extracted timestamps may correspond to the creation time if the item has never been modified or to the last modification time if changes occurred. Most EHR^e^ systems do not maintain a full history of modifications, and even when they do, this information is rarely extracted. For example, laboratory results go through multiple updates: preliminary result, updated preliminary result, validation by a technologist, and final validation by a biologist. While the result value rarely changes, modifications are possible, and updates typically occur within minutes or hours. In contrast, demographic data, such as a patient’s home address, may change over months or years. As only the most recent address and its last modification timestamp are usually extracted, historical changes might be missing. If spatial exposures are relevant to a prediction task, and extracted data spans over multiple years in which changes likely happened, important information might be missing. Recommendation: extraction documentation should clearly reflect the meaning of extracted timestamps, for example, creation, modification, or result validation time. Insights from system users on how frequently modifications occur can help researchers determine whether the extracted data accurately reflects historical records. METRIC: timeliness - currency; documentation; WW: currency.
No date or timestamps available	Description: some items are not extracted with a timestamp or have dates but no time. For example, ICD^f^ codes are linked to admission and usually not timestamped; pharmacy medication orders have a date associated, but no timestamp. Recommendation: a conservative approach is to encode the timestamp at the end of the relevant time unit (eg, ICD codes at the end of the admission [3] and medication orders at the end of day) or a less conservative approach by assigning a typical time of registration (eg, a very high percentage of medications are ordered before noon, assign 12:00 PM). METRIC: timeliness - currency; WW: currency.

^a^METRIC: Measurement Process, Timeliness, Representativeness, Informativeness, Consistency Cluster and Dimension Mapping.

^b^WW: Weiskopf and Weng dimension mapping.

^c^AKI: acute kidney injury.

^d^CLABSI: Central Line-Associated Bloodstream Infections.

^e^EHR: electronic health record.

^f^ICD: International Classification of Diseases.

**Table 9:** Feature engineering recommendations (time-sensitive) related to temporal leaks and timing errors.

Problem	Description and recommendation
The extracted timestamp does not reflect the time when the data are recorded in the system.	Description: the precise timestamp when items are recorded (or even modified) in the system might not be extracted, or might not be stored in the EHR^a^ database for extraction. Typical timestamp differences occur for nurse and clinician observations and coded procedures. For example, nurses might record observations in the patient chart after they finish a shift, and they might record an approximation of the time they observed the patient, for example, at 10:13 AM, the nurse inserts a wound care observation with the time of observation: 8:20 AM (an estimation of the time the nurse visited the patient). Coded procedures (CPT^b^ codes), such as catheter insertion, can be recorded at or after the time of the procedure’s completion, and the timestamp assigned to the procedure is the execution time or a close approximation of it. However, CPT codes can also be reviewed and documented retrospectively, even after patient discharge, when coding is reviewed for billing, based on the patient’s documentation throughout the hospitalization. There can be variation in the recording process, even within the same procedure code: sometimes procedures’ codes are recorded at the time of execution, and other times after patient discharge. The discrepancy between the data available at model development and the data available at prediction time in the clinical implementation may be larger for coded procedures compared to the nurse tasks, because of their review for billing purposes. Recommendation: the extraction documentation should mention the meaning of the extracted timestamp and its usual recording procedure, and possible delays in recording. These are, though, difficult to document exhaustively, as the nurse flow might differ in different wards and coding procedures might vary widely, situations in which understanding of the data recording procedures will require communication with the clinical experts. METRIC^c^: timeliness - currency; documentation; WW^d^: currency.
Exact-hour timestamps often do not reflect the time when data are recorded in the system	Description: if a large proportion of timestamps in a date or time field appear as exact hours (eg, 08:00 AM or 11:00 PM), it may indicate that the extracted timestamps deviate from the time of recording in the system. Nurses might complete the tasks while they are at the patient’s bedside (09:12 AM), but they record them in the EHR software after they finish their round (at 11:31 AM), defaulting the execution time to a preset planned time, such as the start of a nurse’s shift or a standard scheduled time (08:00 AM), instead of the time the task was executed in the system. In this example, the extracted timestamp might be 08:00 AM, while the time available in the system for prediction would be 11:31 AM. Depending on the EHR software and the hospital flow, an exact-hour timestamp could also mean that the task was planned, but not executed (when task execution status is not available in the extraction). Recommendation: the data extraction documentation of the extracted timestamp columns should mention the exact meaning of the extracted timestamps: execution, planning, or registration time, or a combination thereof. Although the procedures for data recording might differ from ward to ward, whenever possible, the hospital flow should be documented and understood. When the exact-hour timestamps do not represent the meaningful timestamps, but the clinical items recorded are of predictive value, encoding the timestamps at the end of the relevant time unit (eg, end of nurse shift) for model training would help in avoiding temporal leaks. METRIC: timeliness - currency; documentation; WW: currency.
Temporal leaks due to extraction errors	Description: temporal leaks can also occur because of errors during the ETL^e^ process, for example, introduced by incorrect time zone handling, incorrect joins, or time-based filtering clauses. These are typically difficult to spot during data preparation. If the temporal leaks have a profound effect on the prediction performance, features that are not expected to have very high importance might appear as most important (eg, history of bacteremia being the most important feature in predicting bacteremia during current admission might point to incorrectly leaking the current admission in the extraction of the historical feature). Some measurement timestamps may fall outside the admission period, occurring either before admission or after discharge [20]. While a small number of such cases can be normal in the workflow (registrations carried out on preadmission or discharge decision before a laboratory result is returned by the laboratory), a large number of such cases might point to date shifts during extraction. Generally, temporal leaks will impact the model’s performance, with the impact varying depending on how much time has been leaked. Recommendation: the primary way to safeguard from errors is rigorous testing of the ETL process. Manually checking a random sample of the admissions for the sequence of events [3] (eg, items extracted from ICU^f^ system being present before ICU admission) can detect problems not detected during ETL testing. Building a tentative model on the training data and inspecting feature importance can reveal high-impact temporal leaks. METRIC: timeliness - currency; WW: currency.
Temporal leaks due to incorrect date or time handling, or incorrect linking during data preparation	Description: incorrect time zone handling, incorrect joins, time-based filtering, or grouping and ordering items temporally can introduce temporal leaks during data preparation. Such leaks can distort predictions by providing information that would not be available at the time of prediction in clinical implementation. For example, misalignment of time zones may shift timestamps incorrectly, making future events appear as if they occurred earlier. Incorrect joins between tables (eg, linking laboratory results to patient records without proper date or time filtering) can introduce data that should not be available at prediction time. Recommendation: thorough unit testing of the data preparation pipeline is essential to prevent temporal leaks. For example, when predictions are generated at specific triggers (every 6 or 24 hours), testing should ensure that processing a full admission retrospectively in the data pipeline produces the same results as processing data incrementally up to each trigger time. This step helps confirm that only information available at a given prediction time is used, preserving the model’s real-world applicability. METRIC: timeliness - currency; WW: currency.

^a^EHR: electronic health record.

^b^CPT: Current Procedural Terminology.

^c^METRIC: Measurement Process, Timeliness, Representativeness, Informativeness, Consistency Cluster and Dimension Mapping.

^d^WW: Weiskopf and Weng dimension mapping.

^e^ETL: extract, transform, load.

^f^ICU: intensive care unit.

## Data Cleaning

The data cleaning process consists of identifying and correcting data issues that could negatively impact the performance or applicability of prediction models. Errors in EHR data can arise from manual entry mistakes, improperly connected or malfunctioning devices, or bugs within the EHR software. Such errors are typically uncovered during data exploration and addressed during data preparation. Tools such as the Data Quality Dashboard [37] have been developed to support and streamline the data cleaning process. Artifacts can also be introduced during data extraction or data preparation. Unit tests for both the ETL process and the data preparation pipeline can safeguard against introducing additional errors.

Overzealous correction of errors, such as manual correction during data preparation of every error encountered, might not necessarily prove beneficial for prediction tasks when such corrections cannot be programmatically reproduced during model implementation in clinical settings. This discrepancy can lead to a situation in which training data accurately represent the true patient state, while real-time predictions rely on erroneous data, resulting in reduced predictive performance. Although measures can be taken to address common and documented errors from previous studies or identified during data exploration (Tables 10 and 11), it is impossible to anticipate and guard against all future errors. For example, new EHR software versions and updates may introduce new bugs, even as older bugs are resolved.

**Table 10:** Data cleaning recommendations related to outliers and encoding issues.

Problem	Description and recommendation
Nonnumeric values in a numeric field	Description: nonnumeric values in a numeric field can encode information about the approximate numerical value. Discarding these (or implicitly transforming such fields to numeric and automatically creating missing values) would result in loss of information. A typical example is laboratory values that are capped at a specific threshold due to the detection limit of the assay, meaning measurements beyond this value are not precisely quantified [38]. For instance, D-dimer results may be reported as “>7650” when they exceed the assay’s measurable range. Recommendation: the capped value should be extracted as is and preserved as numeric during data preparation, as it contains more information than a missing value. METRIC^a^: measurement process - device error - accuracy; WW^b^: correctness.
Values outside a physiologically plausible range (outliers)	Description: manually entered numerical values can contain errors. The amount and magnitude of errors depend on the field definition in the EHR^c^ software; for example, some software might not allow values of body temperature higher than 45 °C or dangerous drug doses, displaying an error or warning on the screen when recording such values, while others will allow any numerical value. Recommendation: exploration of feature ranges and outliers should be carried out during data preparation. For all numerical values (eg, laboratory results and vital signs), a physiologically plausible range can be defined in the data preparation pipeline, and such values can be deleted or replaced with missing values to be imputed at a later stage in the pipeline. This procedure is called outlier capping [3]. We do not recommend manually correcting such errors or applying complex rules that cannot be programmatically reproduced at clinical implementation. METRIC: measurement process - device error - accuracy; measurement process - human-introduced error - outliers; consistency - logical consistency - plausibility; WW: correctness; plausibility.
Implausible or inconsistent values within a time series	Description: numeric measurements within a patient over time constitute a time series, and outliers can be explored in the context of previous or future values of the time series. For example, slipped sensors used in ICU^d^ [28], as for example a slipped bladder catheter, can record body temperatures closer to room temperature for a period, for example, 32°C. While such values are physiologically valid for patients on targeted temperature management, they can be implausible in a specific patient context if they occur only for some minutes or hours. Another example constitutes a heart rate of 0 used to mark admissions not linked to a real patient [30], while in the ICU, a heart rate of 0, within the context of a previous normal heart rate followed by a normal heart rate, can indicate that a CPR^e^ procedure was carried out. Race, sex, and age inconsistencies within a patient but across several admissions can occur, for example, for 1 admission the race is Hispanic, for another, White [20]. Presumably, these should be very rare. Recommendation: corrections within a time series should be carried out during data preparation in a programmatic way (automated, reproducible, and re-executable at prediction time), using only previous values from the same time series or from other features. For the temperature use case, a pragmatic approach can be to use only maximum values of temperatures within a time window and use a large enough time window, such as 24 hours, within which it is unlikely to have only low temperature values, except if the patient is on targeted temperature management. If inconsistencies are rare and a programmatic way of correcting these proves unfeasible, a decision to leave as is might be the best option. METRIC: measurement process - device error - accuracy; measurement process - human-introduced error - outliers; consistency - logical consistency - plausibility; WW: correctness; plausibility.
Character encoding problems	Description: character encoding problems, for example, Ménière disease, can either be recorded as such in the EHR fields, due to a bug in the EHR software (that could have manifested only for a limited period), or can constitute a bug in the ETL process. Recommendation: character encoding consistency in the ETL process should be ensured by unit or integration tests. Whenever the problem manifests in the EHR itself, correct mapping to the correctly encoded terminology should be foreseen in the data extraction or preparation. METRIC: measurement process - human-introduced error - carelessness; consistency - rule-based consistency - syntactic consistency; WW: correctness.
Inconsistent or incorrectly specified units	Description: units for height, weight, laboratory results, and other numerical data [20,25] can differ for different measurements, for example, height in centimeters or in meters in function of the patient’s age, or over time, for example, blood glucose in mg/dL and mmol/L. In some EHR systems, units might be specified but might not correctly reflect the units used historically in past years of the data (if the relational database does not store a history of the linked units). Recommendation: whenever units are correctly specified for all measurements, unit conversion can easily be applied during data preparation. Historical units inconsistencies can be detected by exploration of statistics over time (eg, minimum, maximum, and mean values) and corrected only for historical data (assuming the units recorded now will be maintained in future data, at implementation). METRIC: measurement process - source credibility - traceability; consistency - rule-based consistency - syntactic consistency; WW: correctness.

^a^METRIC: Measurement Process, Timeliness, Representativeness, Informativeness, Consistency Cluster and Dimension Mapping.

^b^WW: Weiskopf and Weng dimension mapping.

^c^EHR: electronic health record.

^d^ICU: intensive care unit.

^e^CPR: cardiopulmonary resuscitation.

**Table 11:** Data cleaning recommendations related to missing data and logical inconsistencies.

Problem	Description and recommendation
Clinically implausible combinations of patient characteristics	Description: even when values are within a plausible range for the entire cohort, they can be implausible for the specific patient. Examples include: length of 1 m for a neonate; weight of 200 g for an adult [24], patient age at admission is 50 years, but admission type “newborn” [20], pediatric conditions for adults [37]. Such examples will occur mostly in manually entered data. Recommendation: exploration of feature ranges should also be performed within relevant subgroups for which the plausible ranges differ significantly. As EHR^a^ data extraction includes a rather large sample size and these errors are rare, these can be set to missing and imputed at a later stage, provided that the imputation model uses relevant features in imputation (eg, imputing length using weight and age); mean or median imputation (overall population) might prove as error-prone as the initial error. Manual correction of such errors case-by-case would not provide a consistent correction strategy at model implementation time. Correction schemes that can be programmatically reproduced (such as imputation) are preferred. METRIC^b^: measurement process - human-introduced error - carelessness; consistency - logical consistency - plausibility; WW^c^: correctness; plausibility.
Syntactic variability for missing values	Description: unknown values at the time of registration can be either left as an empty field, or entered as a free text with various values, such as “not mapped,” “unknown,” “not specified,” “N/A,” “NA,” “?,” etc [20,25]. Recommendation: the values entered in a field should be explored, and all values that represent an unknown value at the time of recording should be encoded as missing. METRIC: measurement process - human-introduced error - noisy labels; consistency - rule-based consistency - syntactic consistency; WW: correctness.
Missing values that can be fully recovered from other fields	Description: some extracted data fields might encode, for specific values of the fields, similar or identical information. Admission ward, admission source, and admission type are such examples. Missing values in one of these can, in some cases, be recovered from the other extracted fields. Miao et al [20] encountered a situation of a trivial imputation scenario, in which the admission source was the emergency room, but the admission type was missing. In this case, the admission type is also emergency. Recommendation: whenever the imputation is trivial and easy to reproduce programmatically, this can be performed during data preparation. At the same time, a powerful missing data imputation algorithm that effectively uses patterns in other features might be able to detect and correctly impute such values, given that enough correct examples exist in the extracted data. METRIC: measurement process - completeness; WW: completeness.
Implausible missingness rates	Description: while EHR data will inherently exhibit missing values, missingness can also be an artifact of errors during data extraction or preparation. For dynamic prediction models, missing data patterns can be explored using an aggregation unit that is meaningful for communication with clinical experts, such as a patient day, even if the final model uses a different aggregation time window. This is typically carried out after feature engineering, and can, in certain situations, reveal feature engineering problems. For example, a missingness rate of around 30% for patient age (which is known in EHR for almost all patients) can have as a root cause a problem during linking identifiers, or even in the ETL^d^ anonymization process. No missing data in features in which missing data are expected can be due to an unintentional imputation during the ETL process. Missing data can also occur because of incorrect mappings during extraction. For example, if missingness rates in vital signs in the ICU, where these are measured at the highest frequency, are higher than in other wards, this could prove to be due to incorrect (or omitted) mapping during data extraction or preparation. Recommendation: whenever the missingness rate is not in line with EHR capabilities or with the expected recording frequency in different wards or patient subgroups, the extraction and preparation logic of those features should be verified for errors. METRIC: measurement process - completeness; WW: completeness.

^a^EHR: electronic health record.

^b^METRIC: Measurement Process, Timeliness, Representativeness, Informativeness, Consistency Cluster and Dimension Mapping.

^c^WW: Weiskopf and Weng dimension mapping.

^d^ETL: extract, transform, load.

## Discussion and Conclusion

In this work, we highlighted challenges and recommendations for the extraction and preparation of EHR data for predictive modeling. Adhering to the “garbage in, garbage out” principle, prediction models rely heavily on the quality and relevance of the input data to generate meaningful and reliable predictions. High-quality data extraction and preparation processes can support prediction models with clinical utility. While it is often argued that EHR “data are not collected for research purposes” [21,26-28,39,40], the fact that these data are sufficient for supporting patient care suggests they could be adequate for developing prediction models. Even though the list of challenges may seem extensive, it should not discourage researchers new to working with EHR data. Not all challenges will apply to every project, and as EHR systems continue to evolve, many issues might affect only historical data and not current data at implementation time. Successfully leveraging EHR data requires both an understanding of its limitations and an appreciation of its potential. Despite its complexities, EHR data offer a rich, comprehensive source of real-world clinical information that can drive impactful research and improve patient outcomes. Applied prediction models can exploit the comprehensive clinical information that is not always readily available to all health care professionals, such as nurses, doctors, hygienists, and other therapists, and have proven practical applicability [5].

Data extraction and preparation for predictive modeling using EHR data are resource-intensive processes, with time and cost varying depending on the maturity of the extraction framework, the prediction task, and the team’s experience in working with EHR data. It is estimated that this phase (which generally includes collaboration with data extraction engineers and clinical experts) takes up to 3 months for an entire team [3], but such estimations will be highly dependent on the maturity of the extraction process and of the team’s experience with EHR data. Data quality often varies significantly across EHR systems and extraction processes, as noted by Weiskopf and Weng [23]. Issues such as data gaps, temporal leaks, incorrect linking, inconsistencies in clinical concept and terminology mapping can affect the quality of extracted datasets and compromise the model’s performance and applicability. To address such challenges, we proposed a list of practical recommendations informed by our experiences with EHR data and insights from published studies. We organized the challenges and recommendations into: cohort definition, outcome definition, feature engineering, and data cleaning; the first 3 categories can also be consulted when planning a project. A clear definition of the prediction task or research question, of the intended use, and the intended users of a prediction model are the first critical steps for defining the outcome, the cohort, and the features of interest [27,31]. It is not uncommon to deem the EHR data inadequate for the prediction task before proceeding with the model-building phase [21].

For cohort definition, we recommend extracting a broader patient context beyond the immediate focus, assessing the completeness of data used for inclusion or exclusion criteria and its availability at prediction time, preventing omissions or duplications, and carefully defining episodes of interest for prediction. Outcomes can be derived in different ways, each with advantages and shortcomings. We generally advise against the use of *ICD* (*International Classification of Diseases*) codes (as these can be up- or undercoded and are generally not timestamped), unless carefully assessed as appropriate for the prediction task. A good understanding of the hospital’s processes, thorough verification of outcomes derived in code, manual inspection of labels, and agreement between data sources can also prevent incorrect outcome definition. Mapping terminology to coding systems (eg, *ICD*, Current Procedural Terminology, LOINC, Systematized Nomenclature of Medicine Clinical Terms, or others) can facilitate feature engineering. However, not all items in the EHR system are aligned with standardized terminologies (eg, LOINC codes for laboratory results are often not used). High-quality data extraction documentation and a good understanding of the underlying clinical concepts and health care processes are essential to support feature engineering. Data exploration can reveal unaddressed problems and further inform the construction of meaningful features. For feature engineering in prediction settings, the timestamp when a clinical item is available in the system is of greatest interest, as this is the time at which predictor values become available for prediction in clinical practice. Good documentation and correct interpretation of the extracted timestamps will prevent temporal leaks. Thorough verification of the extraction and preparation processes (using manual or automated tests), data exploration, and reproducible data cleaning can safeguard against data quality issues.

Clinical assessment of the relevance and the sequence of extracted features and outcome within a patient admission by manual verification of a random sample of admissions can further help detect problems [3]. The solutions to specific issues can be implemented at either the extraction or preparation stage. Applicable to both data extraction and preparation are a good understanding of the underlying data structure and current and historical health care processes, collaboration with relevant experts in conducting the work [3,31], and ensuring a qualitative process of extraction and preparation, supported by unit tests. We recommend maintaining consistency in data extraction and preparation between model training and clinical implementation. Exceptions might, though, exist for correcting historical data problems that are not expected to recur in future data.

We hold the opinion that, in the context of prediction models, the extraction process should not attempt to correct errors residing in the EHR database in an attempt to align the data to the clinical reality, but it should reflect the information from the EHR, presented in a simplified format. We recommend that data cleaning steps be performed during data preparation, pragmatically and programmatically, so that they can be reproduced at implementation time. Extracting and preparing the training data in a different manner than for clinical implementation (eg, temporal leaks) poses the risk of potential overoptimistic evaluation, in the light of which the model’s performance when implemented in clinical practice will be lower than expected. We acknowledge that there are divergent views on this topic and that, in the context of inferential studies, when the analysis does not need to be reproduced on future data, corrections during data extraction might be preferred. At the same time, multipurpose extractions (for both prediction and inferential studies) pose the challenge of solving this divergent view.

We do not provide recommendations for correcting (historical) biases in EHR data (eg, racial, gender, and socioeconomic). Research on preprocessing to enhance fairness has mainly focused on weighting and sampling techniques, while our work focuses on data extraction and feature engineering. Identifying and correcting bias typically requires analyzing model performance and comparison across subgroups, which happens after the data are prepared and the model is built. While the primary focus of this tutorial is methodological, several of our recommendations, such as those concerning cohort definition strategies, implicitly reflect data governance considerations, including the need for ethical approval and proper access control. For example, we encourage defining cohorts during data preparation, provided appropriate ethical approvals are in place to extract broad datasets.

While the level of detail for reporting data extraction and preparation in published prediction studies varies, we do not provide specific recommendations on this aspect. Some researchers advocate for comprehensive documentation of these processes [30,41,42], while others emphasize the importance of sharing the data preparation code to ensure transparency [43,44]. Data extraction for public datasets is generally documented in a separate publication. This can be complemented by sharing the data preparation code and describing in the main paper the key differences between the raw extracted data and the final prepared dataset [30]. Each strategy has its advantages, depending on the audience, journal requirements, and the desired trade-off between transparency and conciseness. While we advocate for transparent reporting, this paper does not specifically address reporting guidelines for data extraction and preparation.

Implementing terminology mapping and standardization during the data extraction phase (eg, through OMOP CDM) can significantly reduce the effort required during data preparation and enable research on multisite datasets extracted from different EHR systems. Furthermore, toolsets for OMOP CDM EHR data exploration and quality assessment have been developed as part of the Observational Health Data Sciences and Informatics initiative, such as Achilles [45] and Data Quality Dashboard [37], which facilitate more than 3500 data quality checks. While we recommend standardization as a solution for some of the listed challenges, we aimed to cover both standard and nonstandard extractions, as the adoption of the OMOP CDM framework may involve higher costs and effort, which can be prohibitive for some hospitals.

Our work has several limitations. First, it is based on our experiences and a selective literature review that cannot be exhaustive. We acknowledge that every project will face use-case-specific challenges. Insights from other research groups working with EHR data would likely highlight additional challenges and recommendations that we may have overlooked, offering a more complete set of recommendations. Second, our focus was on single-site structured EHR data. Extensions to multicenter datasets or unstructured data are possible. Multicenter datasets pose additional challenges with regard to aligning clinical concepts and following the same patient in different hospitals or general practitioner systems. Patient care happens across multiple systems, and single-site extractions provide only a fragmented view of the entire patient care. Third, we provide recommendations for problems that can impede the model implementation in practice, but we do not explicitly cover post implementation challenges, although some of our recommendations can inform on monitoring checks that can be implemented, which remains a subject for medical device post market surveillance. Fourth, while we emphasize the importance of high-quality data extraction and preparation as the foundation of reliable predictive models, we do not assess the impact of each challenge on the final prediction model. The impact will likely depend on the magnitude of the problem, the subgroups in which it manifests, the prediction task, or even the type of model (eg, tree-based models are generally resilient to outliers). While the impact of dataset size, missing data, and outcome definition on the model performance has been studied [46], the impact of other steps in the data preparation procedure remains unknown. Researchers identify and resolve problems during data preparation without assessing their impact on model performance, which we acknowledge having done the same. Research on measurement error [47] demonstrates that inconsistencies in predictor definitions between training and test data can affect model calibration. A similar impact may occur if correction strategies differ between model training and clinical implementation. Finally, although we focused on models with clinical applicability, by recommending an implementation-ready process to avoid discrepancies between the training data and future data due to data extraction and preparation, we did not specifically address model generalization to new hospital settings. The requirements for reproducing the data extraction and preparation can vary significantly based on the EHR software in use (whether from the same or different vendor), the data extraction platform, and the hospital workflow and data registration procedures. Sendak et al [29] estimate “approximately 75% of the effort invested in the initial data preparation for developing prediction models must be reinvested for each hospital.” We argue that the estimation would vary widely, with standardized extractions from the same EHR software potentially requiring less time. Further research could focus on extending the list of challenges and recommendations based on the experience with EHR data of other research groups, extending the scope to larger extraction contexts, or assessing the impact of erroneous or suboptimal extraction and preparation on the final model. We strongly encourage researchers to conduct such impact studies, as this remains a notable gap in the current literature. Understanding how problems propagate through the pipeline is essential for developing trustworthy and clinically meaningful prediction models. We also recognize that decisions made during data extraction and preparation have direct implications for patient safety. Model transparency, reproducibility, and clinical accountability begin early in the pipeline; poor documentation or inconsistent preprocessing can lead to silent errors that affect downstream predictions. Future work could further explore how data extraction and preparation decisions impact not just model performance but also the safe and ethical use of prediction models in clinical settings.

The extensive list of challenges and practical recommendations for EHR data extraction and preparation presented here is intended to improve the quality of research and the practical applicability of clinical predictive models. As all modeling efforts begin with the underlying data, failing to address data quality issues risks producing unreliable and nongeneralizable models. Our focus extends beyond the initial data curation stage in the artificial intelligence life cycle [48]; we also address early-stage issues that can ultimately negatively impact model deployment. Recognizing that there is no “one-size-fits-all” solution, our list of challenges and recommendations, though not exhaustive, is comprehensive enough to support many EHR-based prediction projects. Implementing the strategies applicable to each project can ultimately enhance robustness, reproducibility, and real-world impact of EHR-based prediction models.

## Appendix

Multimedia Appendix 1 Use case and glossary.

## Abbreviations

CLABSI: central line-associated bloodstream infections
EHR: electronic health record
ETL: extract, transform, load
ICD: International Classification of Diseases
ICU: intensive care unit
LOINC: Logical Observation Identifiers, Names, and Codes
METRIC: Measurement Process, Timeliness, Representativeness, Informativeness, Consistency
OMOP CDM: Observational Medical Outcomes Partnership Common Data Model
RESTful: Representational State Transfer
