# Supplementary material for: Challenges and Recommendations for Electronic Health Records Data Extraction and Preparation for Dynamic Prediction Modeling in Hospitalized Patients: Practical Guide and Tutorial
Source: J Med Internet Res. 2025 Oct 17;27:e73987. doi: 10.2196/73987 (PMC12579287; doi:10.2196/73987)
Supplement: Multimedia Appendix 1 [file jmir_v27i1e73987_app1.docx]

# Use case: CLABSI prediction using data from a single-center university hospital

To support our study on developing a prediction model for central line-associated bloodstream infections (CLABSI) [8], we extracted data from UZ Leuven Hospitals, Belgium, for patients admitted between 2012 and 2020. Data from 2012-2013 were used for preliminary methodological studies, while data from 2014-2020 were used for building and evaluating the CLABSI prediction model. The data were consolidated from five primary sources: the hospital's Electronic Health Record (EHR) system, the Intensive Care Unit (ICU) Patient Management System, the Pharmacy Management System, the Laboratory Information System, and the Hospital Information System. These were centralized as multiple “base tables” in a data warehouse and further preprocessed in an R model pipeline. Below, we outline the main challenges and our corresponding solutions. While not exhaustive, the challenges discussed below are those we subjectively identified as the most significant in shaping our study design.

**Cohort definition.** Our cohort consisted of patients with at least a central catheter: centrally inserted central catheter (CICC), tunneled cuffed and non-cuffed central venous catheter, port-catheter (Totally Implanted Vascular Access Devices, TIVAD), peripherally inserted central catheter (PICC) and dialysis catheter. This constituted our only inclusion criterion; no exclusion criteria were applied for the main study period (2014 – 2020).

- While exploring temporal trends, we observed that neonates were not present in the first year of data (2012), as the Neonatology department used paper-based records during that year. Consequently, we excluded neonates from our preliminary studies conducted on data from 2012 and 2013 [6,7].
- Catheter information had to be identified and mapped across two different systems: the ICU database and the general EHR used by non-ICU wards. These systems documented catheters differently. In the ICU, catheters had explicit start and end dates recorded in the system by the nurses at catheter insertion and removal. Outside the ICU, catheter data were recorded as nursing observations rather than formal devices with unique identifiers. These nursing observations, e.g. catheter dressing aspect, contained information about catheter type and location. Start and end times were inconsistently registered in the EHR. Since catheter observations were recorded consistently in both systems, we used these to define catheter episodes both in ICU and outside ICU. Additional details on catheter episode definition are available in our study [8].
- We encountered several inconsistencies in catheter registration within the hospital-wide EHR. First, port-catheters were recorded with a code but no name, causing them to be missed in the initial data extraction. Second, dialysis catheters were recorded in the Nephrology department in a different form and table in the database for a part of the study period, which led to dialysis catheters being missed during the initial extraction. These issues required re-extraction of data to ensure that admissions with central catheter are not mistakenly excluded from the cohort. Third, the codes and names of catheters changed over time, and for some periods these were coded and registered with typos, leading to unintentional exclusion of catheters when codes had typos.
- The cohort was defined at the data extraction stage by including only admissions with central catheters. This has limited data available for a hospital admission, e.g. data corresponding to an admission with catheter only in ICU were not extracted from the hospital-wide EHR or vice-versa. Re-extractions and re-definition of the cohort at extraction were necessary.
- Finally, while exploring the processed catheter episodes, we observed fragmentation due to weekend effects, i.e. during weekends, the catheter observations registration compliance was lower than during week days; catheter episodes were ended if no observation was recorded for 48 hours, which sometimes occurred over weekends. Although we evaluated correction strategies, they tended to overcorrect, and we ultimately accepted a certain level of fragmentation.

**Outcome definition.** We defined the outcome as the occurrence of CLABSI, discharge, or death within seven days and we renewed predictions every 24 hours since start of a catheter episode. The extraction of auxiliary outcomes (death and discharge) was straightforward and did not pose challenges. The clinical definition of CLABSI is explained in our main paper and the pseudocode in the supplementary material explains the calculation logic [8]. Briefly, CLABSI refers to bloodstream infections in patients with central catheters, not attributable to other sources and thus presumed to be catheter-associated.

- We considered three approaches to outcome definition: (1) manual assessment by infection preventionists for governmental reporting, (2) ICD codes, and (3) algorithmic derivation from the extracted data. We excluded the ICD-based approach early due to the lack of timestamp information, which is critical for time-sensitive predictions.
- The manually assessed CLABSI records were treated as a reference standard, as they involved clinical judgment and were timestamped. However, these were stored in separate Excel files, and we observed inconsistencies and date shifts when importing into the data warehouse. Moreover, no manual CLABSI evaluations were available for 2012, as the hospital started assessing and reporting CLABSI in 2013.
- We implemented the Sciensano CLABSI definition programmatically. We thoroughly validated it against the manually assessed CLABSI through three rounds of testing together with an infection preventionist and a clinician. Misinterpretations in the algorithmic version were corrected iteratively. By the third round, the remaining discrepancies were due either to human error or logic based on imaging reports, which we didn’t parse in our calculation. The imaging reports exclusions were through rare. We therefore concluded that the derived definition was robust and consistent across all study years. Although complex, our implementation remained pragmatic, e.g., we skipped symptom checks assuming lab tests are only ordered when symptoms are present. The calculation of the CLABSI definition was not affected by missing data.
- We have discussed the risk of label leakage due to temporal leakage. The CLABSI timestamp is derived from the collection time of a positive microbiology sample. Microbiology samples are ordered based on infection symptoms, such as fever. The real time of infection might though be at or before symptoms onset, which is earlier than the defined CLABSI timestamp. This could lead to a model that detects rather than predicts (forecasts) CLABSI, particularly if the model performs better for a two-day prediction horizon rather than a seven-day horizon. Additionally, because CLABSI prevention interventions differ from CLABSI treatment interventions, a detection model would have very limited utility, if any. Sensitivity analyses will be performed if the model is implemented in clinical practice.

**Feature engineering.** Based on input from clinical experts on relevant predictors for CLABSI, we extracted 302 distinct features encompassing patient demographics, ward transfers, medication, laboratory test results, comorbidities, vital signs, symptoms and catheter registrations. We used a feature aggregation window of 24 hours, aligned with the prediction renewal frequency (every 24 hours), with some exceptions: for example, medication orders were aggregated every 3 or 7 days. Additional information is available in our study and the supplementary material [8].

- In the initial extraction, patient age at admission was missing for around 30% of the extracted admissions, which represents an implausibly high rate of missingness for EHR data. This was traced to incorrect linking of identifiers and was resolved through re-extraction.
- Several care items (catheters, scales, scores, signs, symptoms, etc.) were extracted differently depending on their source: the ICU system or the hospital-wide EHR system. Each system had distinct table formats and structures. Denormalization (simplification of table format) was partly carried out during data preparation. Terminology harmonization was carried out fully during data preparation. These tasks required extensive effort to understand the database format, the meaning of the recorded data and the historical changes in data recording patterns (e.g., how documentation in the nephrology department changed over time). This work involved close collaboration between researchers, data extraction engineers from both ICU and non-ICU systems, clinical experts (nurses and physicians for ICU and other wards), and hospital IT staff with in-depth knowledge of the database schema. Clinical concepts were mapped during data preparation using mapping tables stored in a configuration file, enabling the pipeline to be easily adapted to data from other hospitals by simply updating hospital-specific mappings.
- We observed high variability in the way pain scales were recorded across medical wards and age groups. As pain was not deemed a key predictor for CLABSI, we decided not to extract and harmonize these.
- Patient diagnoses were recorded both as ICD codes linked to closed admissions and through clinical pathways during patient’s hospitalization. Clinical pathways generally captured only the primary diagnoses, whereas ICD codes provided broader diagnostic detail. However, ICD codes lacked timestamps and were inconsistently recorded during the hospital’s transition from ICD-9 to ICD-10, resulting in a data gap of almost one year. Since we used diagnoses only as historical comorbidities (e.g., any prior diagnosis of diabetes), and not as time-varying features within catheter episodes, we opted to use ICD codes for their completeness. The effect of the data gap during the transition period was mitigated by aggregating diagnoses across long time windows. Because of the high granularity of ICD codes, these were mapped to Clinical Classifications Software (CCS) categories during data extraction. The aggregation step (diagnoses linked to any of the previous admissions belonging to a patient) was performed during data extraction, as access to all patient admissions was only possible at that stage. In contrast, the data preparation pipeline had access only to admissions with central catheters and was left-censored by the extraction period.
- The exploration of temporal trends in variable recording revealed that certain features (e.g., ciclosporin and troponin I) stopped being recorded during the training period. These were excluded from the final feature set as they are known to be unavailable in future data.
- The exploration of catheter episodes revealed that catheters care items were recorded less during weekends. We created separate features for the day of the week (Monday to Sunday) to allow models to learn weekday/weekend effects and interactions with various features.
- For deriving and aggregating laboratory and microbiology features, we used the timestamp of the final validated result, i.e., the time when results became available in the system. In contrast, the CLABSI outcome definition was based on the lab sample collection time, as this reflects the clinically relevant timestamp. Using the sample collection time for feature definition would introduce temporal leaks, as at that time the lab results are not available in the system for real-time prediction.
- We observed that certain nursing care items were recorded using exact hour timestamps, such as 08:00:00 or 23:00:00. These do not represent the actual time of registration in the system, but a generic nurse shift time used for nurse tasks planning. As care items are typically recorded three times a day (during each nurse shift), and our 24-hour aggregation window covers three shifts, we considered the risk of temporal leaks due to these generic timestamps would be minimal.
- Finally, we implemented unit tests during data preparation to verify the correct temporal handling of all features and to safeguard against unintentional leakage of future data. During testing, we identified and corrected several bugs related to incorrect processing of timestamps or incorrect sequence of joining tables and filtering records.

**Data cleaning.** We have performed only minimal data cleaning. All data cleaning actions were based on informal data exploration during feature engineering.

- Special characters in laboratory values (e.g., a value of “≥7650” for D-dimer) were stripped to retain only the numeric component (e.g., “7650”) before converting to numerical format.
- For outlier correction, based on previous experiences of working with highly granular temperature values in ICU, maximum hourly values have been extracted from the ICU database. Further, during data preparation we have used maximum values in time windows of 24 hours. This two-step aggregation during extraction and preparation avoided abnormally low temperature values due to slipped sensors. Further, we have deleted temperature values <30 and >45 centigrade and considered these missing values.
- From our previous experiences of working with highly granular temperature values in ICU, we knew that incorrect outliers (low temperature values due to slipped sensors) will exist and these are difficult to distinguish from correct outliers, due to patient on targeted temperature management. We extracted only maximum hourly values from the ICU system during data extraction. During data preparation we further aggregated this values in 24 hours windows using maximum function as the aggregation rule. This two-step aggregation during extraction and preparation avoided abnormally low temperature values due to slipped sensors. Additionally, we have deleted temperature values <30 and >45 centigrade and considered these missing values.
- Values outside the possible ranges were also deleted for other vital signs: systolic and diastolic blood pressure, respiratory rate, heart rate, oxygen saturation, CVP (central venous pressure), weight, length, glycemia, as explained in the supplementary material of our study [8].
- We did not apply such range filters to laboratory test results, as these are generated by machines and typically less error-prone. However, as noted in our study, the regression model exhibited lower calibration because of extrapolation on extreme D-dimer values, due to a laboratory machine upgrade in 2019.
- The BMI feature contained outliers due to incorrect and implausible weight or length measurements, mostly in neonates. We have discarded the BMI from our feature set, as it was deemed less important for CLABSI prediction.
- We experienced various character encoding problems in multiple data items, both due to data extraction (these were fixed with correction of the extraction process and re-extraction) and to EHR software bugs (these were fixed during data preparation).
- No unit transformations were applied to laboratory or vital sign data, as all extracted items used consistent units during the extraction period. Nevertheless, in the data preparation part of the ML pipeline, we implemented a unit check, and a warning would be issued if the code is run and new units would be encountered.
- We performed formal exploration of missing data patterns across subgroups, such as the medical ward. Surprisingly, ICU showed higher missingness for some vital signs compared to non-ICU wards, while ICU patients are more closely monitored and the missingness rates are expected to be the lowest in ICU. This discrepancy was traced back to incomplete variable mappings during data extraction, which were subsequently corrected.

# Glossary of technical terms

#### Base Table

A table containing data of a specific clinical domain (eg, laboratory results, diagnoses, and medications) extracted from the EHR database. Often aligned with structures used in data models such as OMOP CDM.

#### Capped Values

Values for a variable are limited to be within a range to prevent extreme outliers from influencing the model or slowing down model convergence when building a model.

#### Denormalization

The process of flattening relational data into simpler table formats to facilitate analysis, often carried out during data extraction. EHR databases are mostly relational databases, in which distinct items (eg, laboratory tests and laboratory results) are stored in separate tables and referenced whenever needed. For example, a hemoglobin laboratory test is stored with a code and a name in a table. The results are stored in a separate table referencing the laboratory tests table. When denormalized, the test name (hemoglobin) and test value (13.5) are extracted in the same base table.

#### Dynamic Prediction

A modeling approach where predictions are updated continuously throughout a patient’s hospitalization, either at prespecified intervals (eg, every 12 hours or daily) or trigger-based, where triggers represent an event in the EHR database that initiates a new prediction (eg, as new patient data, such as laboratory values, become available).

#### Episode

A time-bound segment within a patient’s hospital stay that is clinically meaningful for the prediction task. It represents the window of interest during which the patient is considered to be at risk for the outcome being predicted (eg, infection, deterioration, and readmission).

#### ETL (Extract Transform Load process)

A data engineering process for extracting data from relational databases, transforming it into a simplified format, and loading it into a data warehouse.

#### Feature

A variable used as an input for prediction models. In EHR-based prediction models, features include, for example, different laboratory results, medications, or vital signs.

#### Feature Aggregation

Combining multiple raw data points into descriptive statistics, such as mean or maximum, in a time window.

#### Feature Engineering

Transforming raw data points into usable and informative features.

#### FHIR (Fast Healthcare Interoperability Resources)

An international standard for exchanging health care information electronically, often used for real-time data transfer between systems. When implementing a prediction model in clinical practice, it is critical that the model can retrieve patient data in real time (eg, laboratory results, medication, and vital signs) and return predictions to the EHR system promptly and securely. FHIR facilitates this by providing a standardized way for different health care systems and applications to communicate.

#### OMOP CDM (Observational Medical Outcomes Partnership Common Data Model)

The OMOP CDM is a standardized data structure developed by the Observational Health Data Sciences and Informatics community. It is designed to enable the harmonization, sharing, and large-scale analysis of observational health care data across institutions, countries, and data sources (such as EHRs and insurance claims).

It provides a unified data schema. For example, patient demographics, drug exposures, procedures, diagnoses, and laboratory measurements are stored in predefined standardized tables (eg, PERSON, CONDITION_OCCURRENCE, DRUG_EXPOSURE, MEASUREMENT, etc). It also maps local codes and terminology (eg, *ICD-10* [*International Statistical Classification of Diseases, Tenth Revision*], Systematized Nomenclature of Medicine, LOINC, and RxNorm) to a standardized vocabulary, ensuring semantic consistency across datasets from different systems.

#### Label Leakage

An unintentional inclusion of (partial) information about the outcome to be predicted in the input features used for model training and model evaluation, leading to overly optimistic model performance.

#### Prediction Trigger

A defined point in time (eg, every 24 hours, or upon a new laboratory result) when a model is run to generate a new prediction for a patient.

#### Temporal Leak

The unintentional use of information from the future, relative to the prediction time, during feature engineering and model training. This can occur when data that would not be available at the time of a real-life prediction is included in the input to the model. As a result, the model may learn patterns that unfairly boost its performance but would not be usable in practice, leading to artificially high performance metrics. Examples are: using laboratory results timestamped after the prediction point (the model predicts sepsis at noon using laboratory values from 4 PM) or including interventions that occur later in the admission in features used to make earlier predictions (using antibiotic administration given in response to a confirmed infection to predict the infection itself).

#### Unit Testing

A type of software test that verifies that individual pieces of code or functions work as expected. It ensures the correctness of the code written. It should not be confused with model testing or evaluation, which assesses predictive performance.
